# When the Heart Cries Wolf: Myocardial Bridging Presenting as Angina-like Chest Pain

**DOI:** 10.7759/cureus.5392

**Published:** 2019-08-15

**Authors:** FNU Farukhuddin, Muhammad Akrmah, Maryam R Hussain, Aqsa Iqbal, Mahboob Alam

**Affiliations:** 1 Neurology, University Hospital Cleveland Medical Center, Cleveland, USA; 2 Epidemiology, Icahn School of Medicine at Mount Sinai, New York, USA; 3 Cardiology, University of Illinois at Chicago, Chicago, USA; 4 Cardiology, Baylor College of Medicine, Houston, USA

**Keywords:** myocardial bridging, myocardial ischemia, angina, coronary angiography

## Abstract

Myocardial bridging (MB) is the most common congenital coronary anomaly and refers to an intramural course of an epicardial coronary artery. The proximal segment of the left anterior descending artery (LAD) is the most commonly involved vessel and is often seen in patients with hypertrophic cardiomyopathy (HCM). We present a case of a 64-year-old female with left-sided non-exertional chest pain. Electrocardiography (EKG) and echocardiography were negative, however, stress EKG was positive with deep ST-segment depressions. Coronary angiography revealed mid-segment compression of LAD during systole, returning to its normal caliber during diastole. The patient remained asymptomatic during the hospital course and was later discharged on beta-blocker therapy. This case is different from others in a sense that it presented with severe pain like angina and mid-segment of LAD is involved rather than the proximal segment where it commonly occurs. This case report will help clinicians overcome the diagnostic challenge in patients presenting with atypical chest pain.

## Introduction

Myocardial bridging (MB) is the most common congenital coronary anomaly and refers to an intramural course of an epicardial coronary artery. Normally, coronary arteries are present on the surface of epicardium but they sometimes can extend into the myocardium. This intramyocardial segment is referred to as a “tunneled” artery, which appears later on the surface of the heart, giving rise to the term “myocardial bridging” [1]. The proximal segment of the left anterior descending artery (LAD) is the most commonly involved vessel and is often seen in patients with hypertrophic cardiomyopathy (HCM) [2-3].

The prevalence of myocardial bridging is less than 5% when assessed by coronary angiography but this finding is much more common during an autopsy and can be seen in almost 30% to 80% of adults [2]. Myocardial bridging is generally a benign condition and often an incidental finding on coronary angiography but it can also lead to arrhythmia, acute coronary syndromes, myocardial ischemia or infarction, as well as sudden cardiac death [4]. We report here a case of myocardial bridging in a 64-year-old woman who presented with angina-like chest pain.

## Case presentation

**Figure 1:**
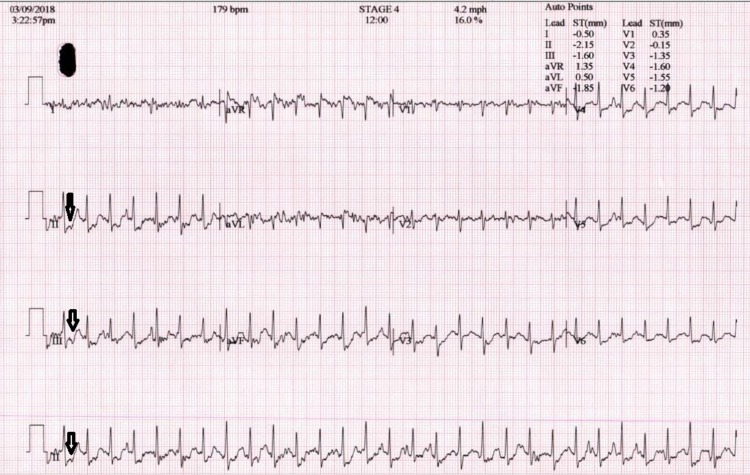
Electrocardiography after exercise

A 64-year-old female with no significant past medical history was seen by her primary care physician for a complaint of left-sided, intermittent, non-exertional chest pain and pressure for two months. Her symptoms were not exacerbated by strenuous activity or exercise but she did experience exertional dyspnea, dizziness, and lightheadedness without a syncopal episode. She was subsequently referred to our cardiology clinic for further evaluation. Echocardiography and a Holter test were unremarkable. A treadmill stress test did not provoke any symptoms but was positive with deep ST depressions (Figure 1).

**Figure 2:**
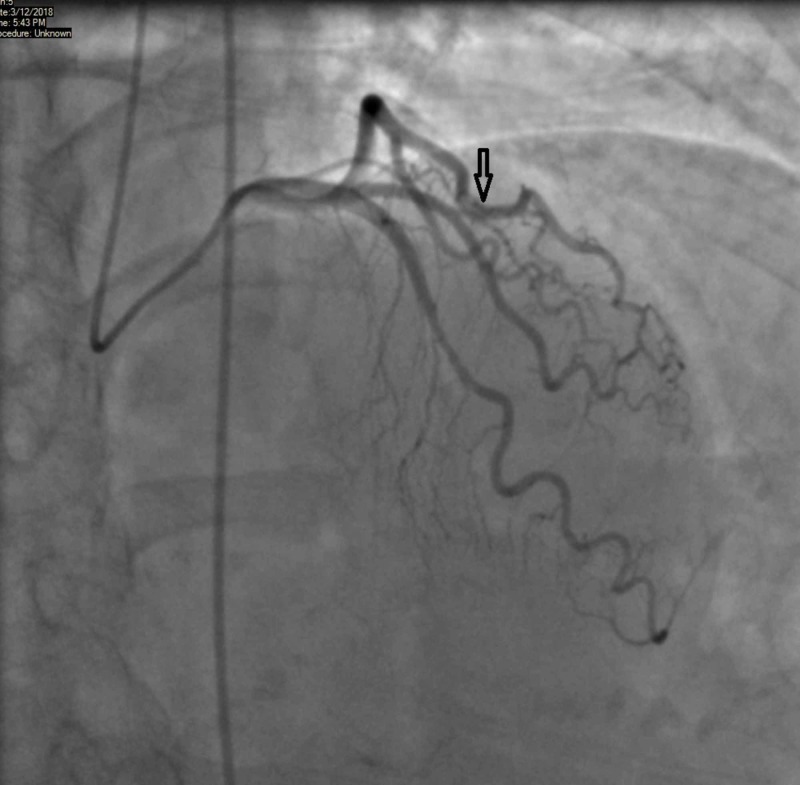
Angiography during systole

**Figure 3:**
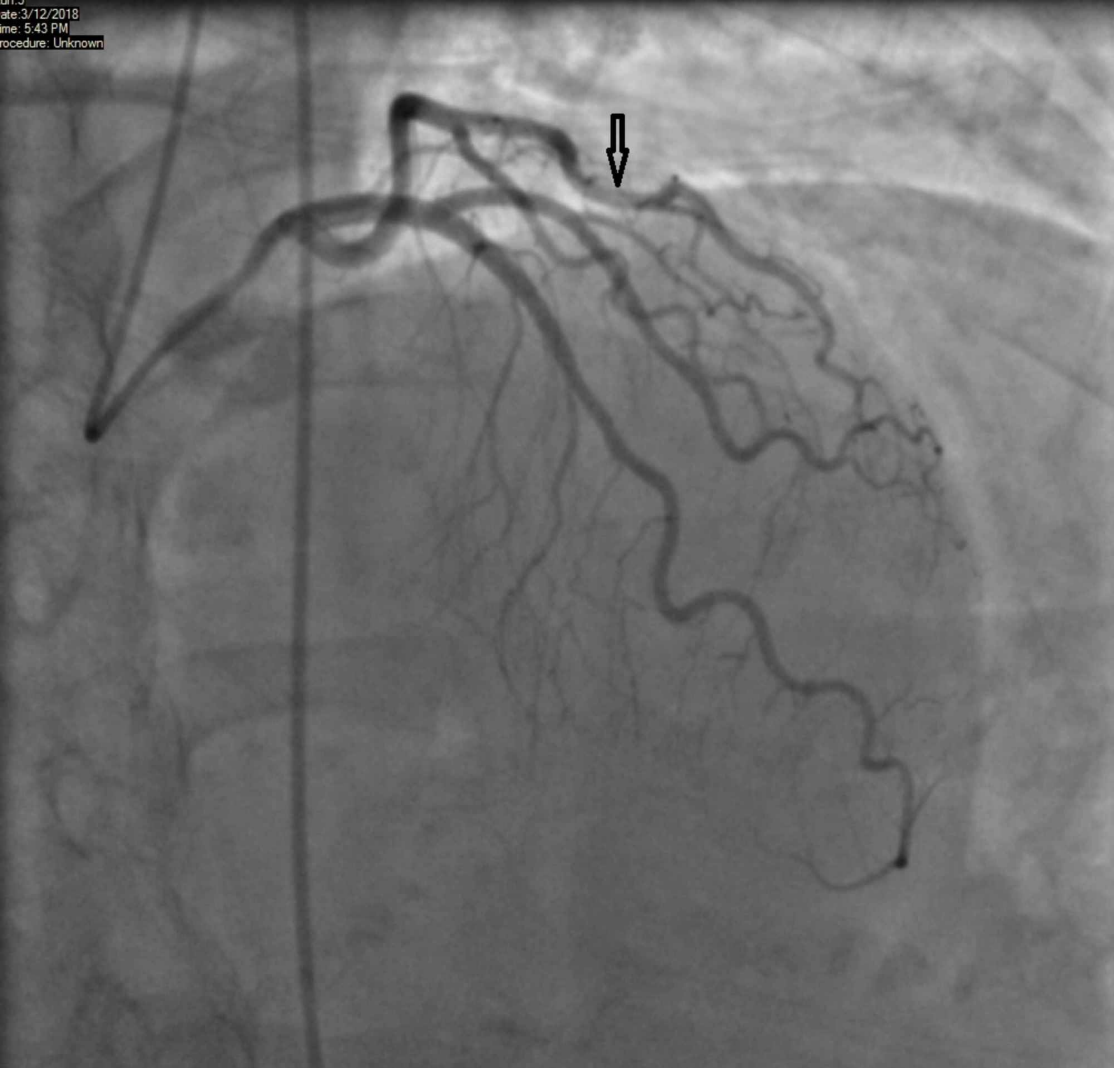
Angiography during diastole

Given her stress test findings, it was decided to admit her for observation and perform a further diagnostic evaluation. Coronary angiography did not reveal any blockage of major coronary vessels. However, to our surprise, we noticed that the mid-segment of the LAD coronary artery was found to be transiently compressed during systole and later returning to its normal caliber during diastole (Figures 2-3).

This finding incurred a diagnosis of myocardial bridging. The patient remained asymptomatic during her course of stay and was discharged home on aspirin and metoprolol. After one month of follow-up, the patient responded well on medications.

## Discussion

Myocardial bridging is usually a benign and rare condition but it is an important differential diagnosis in adult patients presenting with syncope and angina-like symptoms [2-3]. The true prevalence of myocardial bridging is difficult to discern since the method of evaluation can lead to a misinterpretation of findings, subsequently underdiagnosing or over-diagnosing myocardial bridging. There is concordance among the prevalence rate of myocardial bridging as detected by pathological analysis and non-invasive imaging studies, ranging from 5% to 86% [3]. However, angiographic studies have found the prevalence of myocardial imaging to be relatively lower, with studies reporting a prevalence of less than 2% (range 0.5% - 16%) [2-3]. Although theoretically any segment of the coronary vasculature can be effected, the LAD is most commonly afflicted with this anomaly. Besides the systolic compression that was identified in coronary angiography in our patient, studies utilizing coronary angiography have identified several features attributed to myocardial bridging, including an echo lucent half-moon phenomenon that persists throughout the cardiac cycle over the bridging segment [5].

The first-line treatment in symptomatic patients is medical management with beta-blockers and calcium channel blockers [6-7]. Nitrates reduce the coronary wall tension, which in turn increases reflex sympathetic stimulation and contractility. This could propagate the severity of vascular compression during systole, and hence the use of nitrates is contraindicated in the setting of myocardial bridging [8]. Percutaneous coronary intervention is considered in patients who remain symptomatic on medical management. However, the percutaneous coronary intervention has been found to have poor treatment outcome with in-stent restenosis rates of 36% to 75% with bare-metal stents and 18% to 25% with drug-eluting stents [9]. Furthermore, there remains an increased risk of stent fracture, in-stent thrombus, and coronary perforation. Bypass surgery and myotomy are other alternatives considered for patients with refractory symptoms, especially in those with extensive and deep bridges [10]. The patient reported here had no further symptoms and therefore discharged home on aspirin and beta-blockers.

## Conclusions

In conclusion, myocardial bridging is a rare yet important diagnosis and should be considered in patients with atypical angina-like chest pain and diagnostic findings that may be discordant with the patient's symptomatology. Bridge can sometimes involve nonusual segments of the coronary artery as presented in this case.
